# A Novel Fast Photothermal Therapy Using Hot Spots of Gold Nanorods for Malignant Melanoma Cells

**DOI:** 10.3390/nano8110880

**Published:** 2018-10-28

**Authors:** Yanhua Yao, Nannan Zhang, Xiao Liu, Qiaofeng Dai, Haiying Liu, Zhongchao Wei, Shaolong Tie, Yinyin Li, Haihua Fan, Sheng Lan

**Affiliations:** 1 Guangdong Provincial Key Laboratory of Nanophotonic Functional Materials and Devices, School of Information and Optoelectronic Science and Engineering, South China Normal University, Guangzhou 510006, China; m15625107214@163.com (Y.Y.); clzhangnannan@163.com (N.Z.); shawer1004@163.com (X.L.); daiqf@scnu.edu.cn (Q.D.); hyliu@vip.163.com (H.L.); weizhongchao@263.net (Z.W.); 2 School of Chemistry and Environment, South China Normal University, Guangzhou 510006, China; tiesl2008@163.com; 3 School of Life Sciences, Sun Yat-Sen University, State Key Lab for Biocontrol, Guangzhou 510275, China; liyinyin@mail.sysu.edu.cn

**Keywords:** gold nanorods, A375 cells, plasmonic coupling, photothermal therapy, hot spot

## Abstract

In this paper, the plasmon resonance effects of gold nanorods was used to achieve rapid photothermal therapy for malignant melanoma cells (A375 cells). After incubation with A375 cells for 24 h, gold nanorods were taken up by the cells and gold nanorod clusters were formed naturally in the organelles of A375 cells. After analyzing the angle and space between the nanorods in clusters, a series of numerical simulations were performed and the results show that the plasmon resonance coupling between the gold nanorods can lead to a field enhancement of up to 60 times. Such high energy localization causes the temperature around the nanorods to rise rapidly and induce cell death. In this treatment, a laser as low as 9.3 mW was used to irradiate a single cell for 20 s and the cell died two h later. The cell death time can also be controlled by changing the power of laser which is focused on the cells. The advantage of this therapy is low laser treatment power, short treatment time, and small treatment range. As a result, the damage of the normal tissue by the photothermal effect can be greatly avoided.

## 1. Introduction

Gold nanorods (GNRs) are increasingly receiving academic attention due to their special, unique chemical and optical properties in addition to their high biocompatibility [1,2,3,4,5,6]. The size of the GNRs is easily controlled, and by adjusting the size of the GNRs, the absorption peaks are modulated in the near-infrared (NIR) region. Nano-sized particles are also easily taken up by cells. Therefore, GNRs show more and more application potential in photothermal therapy. The longitudinal localized surface plasmon resonance in the infrared region of GNRs renders them good materials for cancer photothermal therapy under NIR excitation. For optics applied in biology, NIR excitation first offers low scattering and energy absorption, secondly offering maximum irradiation penetration into deeper tissue. Moreover, the autofluorescence emitted from nontargeted tissue can be partially or totally inhibited under NIR excitation. The stability and biocompatibility of GNRs also make them suitable be used in biological medicine. The conduction electrons of GNRs can be excited by coherent light to induce surface plasmon oscillations, which can be used for photothermal therapy [7,8,9,10,11,12].

The rapid global economic development has caused major environmental degradation and the destruction of ozone in the atmosphere. More UV penetration increases the incidents of skin cancer. As the largest organ of the human body, the skin is a physical barrier against various infections and environments. Skin cancer is a type of cancer that accounts for almost 40% of the world’s cancer population [13]. Melanoma is one of the most common types of skin cancer. Melanoma (cancer caused in melanocytes, which are the pigment-containing cells) has a highly tendency to metastasize to other organs of the body. Plasmonic photothermal therapy for the treatment of cancer has received a great deal of attention in recent years. Specifically, in the past decade, there has been much progress in the development of GNRs for photothermal therapy applications due to their localized surface plasmon resonance [14,15,16] as well as their inherently low toxicities [17,18,19]. Compared to other cancers, melanoma is mostly located on the surface of the body, making it easy to treat directly with a laser. However, reports of plasmon resonance photothermal therapy for skin cancer cells are lacking. For photothermal therapy, it can be seen that many works focus on the design and modification of photothermal materials. These materials have better properties (such as cell targeting, photothermal conversion efficiency, etc.) and can improve the efficiency of photothermal therapy [20,21]. Some works are focused the effects of photothermal therapy on cells [22]. These studies have led to the further development of photothermal therapy. After summarizing the previous research work, it was found that the range of the traditional photothermal therapy laser action reaches the 2-cm level, the time is generally several minutes to 10 min, and the light source is generally provided by a continuous-wave diode laser. However, there is a lack of research on low-power photothermal therapy on a small scale and in a short period of time. For clinical applications, the energy of the input laser should be as low as possible, the laser treatment range should be as small as possible, and the laser and the biological action time should be as short as possible to avoid damage to healthy tissues. Research on photothermal therapy has been trying to achieving these goals.

In this study, we investigated a low-power rapid photothermal therapy for individual cancer cells by using the plasmonic coupling between GNRs. The cell viability of human malignant melanoma cells (A375) cells still exceeded 80% after incubation with GNRs for 24 h by controlling the concentration of GNRs in the culture medium. This result indicates that the cytotoxicity of GNRs is negligible in the doses used in this work. The transmission electron microscopy (TEM) image of A375 cells shows that the GNR clusters form naturally in the organelles of cells. The simulation results show that the plasma coupling between GNRs can effectively enhance the electromagnetic field in interparticle gap space, known as the hot spot. Therefore, a low-power femtosecond laser can result in rapid acute damage to individual cancer cells by using the hot spot. Necrosis can be induced in A75 cells immediately after they are irradiated by a femtosecond laser as low as 14.6 mW in 20 s. The cell’s death time can also be controlled by changing the power of the laser which is focused on the cells. In this therapy, the laser can directly illuminate the skin cancer cells. The power of the irradiation is very low, the irradiation time is very short, the treatment is small in scope, and it can be controlled to treat only individual cells. As a result, the damage of the normal tissue by the photothermal effect can be greatly avoided. This work represents a big step forward in the efforts to employ the plasma photothermal effect in the actual treatment of disease.

## 2. Materials and Methods 

### 2.1. Synthesis of PEG-Coated GNRs

In this work, the modified seedless method, which is described in detail in Reference [23], was used to synthesize GNRs. The as-prepared GNRs were then stabilized by polyethylene glycol (PEG) [24].

### 2.2. UV-Vis-NIR Absorption Spectroscopy

The absorption spectra of the GNRs were obtained using an ECAN microplate reader (Tecan, Durham, CA, USA). The solution was put into a quartz cuvette. The absorption spectrum of deionized water or PEG was detected, to be used as a baseline to calibrate the spectra of all the samples.

### 2.3. Transmission Electron Microscopy

The morphology of the GNRs was examined using transmission electron microscopy (TEM). The TEM images were taken by using a high-resolution TEM (JEOL-JEM-2100HR, JEOL Company, Akishima, Tokyo, Japan) operating at an accelerating voltage of 200 kV. The samples were prepared on 200-mesh copper grids.

### 2.4. Cell Culture and Cell Viability

The cancer cells used in the photothermal therapy were human malignant melanoma (A375). They were obtained from the Cell Lab of the Cell Resource Center of the Chinese Academy of Sciences. The cell culture methods are very similar to those described in Reference [25].

Cell viability was measured using the 3-(4,5-dimethylthiazol-2-yl)-2,5-diphenyltetrazolium bromide (MTT) assay. This method is similar to those described in Reference 25. The 96-well plate was then put into an iMark Microplate (BioRad) to measure optical densities (OD) at 490 nm. The cell morphology of A375 cells incubated with GNRs at different concentrations are shown in Figure S2.

### 2.5. Cellular Uptake of Gold Nanorods

The concentration of Au was measured by inductively coupled plasma-mass spectrometry (ICP-MS) (ICAP-qc, Thermo Fisher, Boschstr, Kleve, Germany) [26]]. The number of gold nanorods in a single cell can be obtained by dividing the ICP-MS result by the cell density in the ICP-MS sample and then dividing by the mass of a single gold nanorod. The cellular uptake images were examined using TEM observation. After incubation with the GNRs for 24 h in a humidified incubator (37 °C, 5% CO_2_), the cells were washed three times with Phosphate Buffered Solution (PBS) and pelleted by centrifugation. Finally, they were fixed in glutaraldehyde (2.5%), embedded in resin, cut to ultra-thin sections, stained by osmic acids, and finally imaged using TEM at an acceleration voltage of 120 kV. The samples were prepared on Ni mesh with a carbon support film.

### 2.6. Photothermal Therapy Experiments

The schematic diagram of the experimental setup is shown in Figure S3. In the photothermal therapy experiment, a Ti:sapphire oscillator (Mira 900 S, Coherent, Santa Clara, CA, USA) with a repetition rate of 76 MHz was reflected into an inverted microscope (Axio Observer A1, Zeiss, Santa Clara, CA, USA) and was focused on the targeted cell using a 60× objective lens. The diameter of the laser spot was estimated to be ~1 μm. The photothermal therapy experiment was also carried out using a confocal laser scanning microscope (TCS-SP5, Leica Oberkochen, Germany,). For the photothermal therapy experiment, the cells were incubated with the culture medium containing 78 pM GNRs for 24 h. After that, the culture medium containing GNRs was removed and the new culture medium containing no GNRs was added into the dish. The laser power used for the photothermal therapy ranged from 0 to 30 mW.

### 2.7. Numerical Simulations

The finite-difference time-domain (FDTD) software developed by Lumerical Solutions, Inc. (Thurlow Street, Vancouver, BC, Canada, http://www.lumerical.com) was employed to simulate the distribution of electric field of GNRs [27,28]. In the FDTD simulations, non-uniform grids with the smallest grid of 0.2 nm and perfectly matched layer conditions were employed.

## 3. Results and Discussion

### 3.1. Characteristic of GNRs

**Figure 1:**
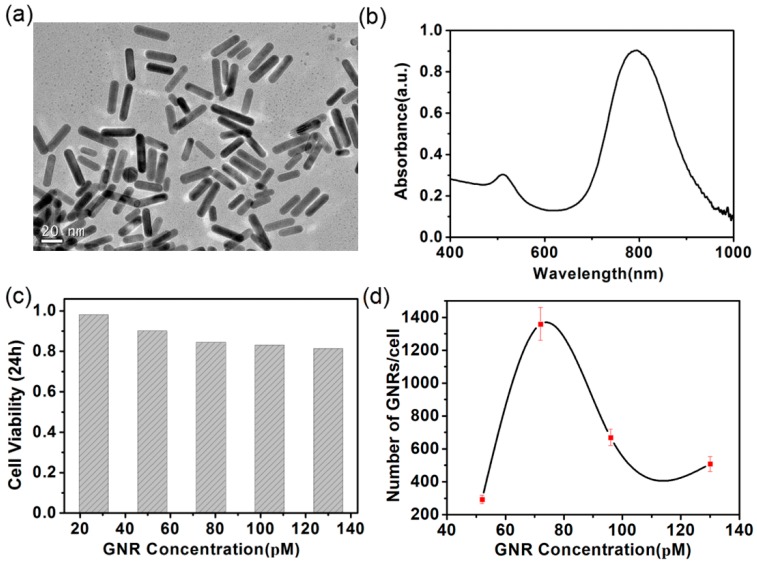
(**a**) TEM image of the synthesized gold nanorods (GNRs); (**b**) absorption spectrum of the GNRs dispersed in water; (**c**) cytotoxicity of GNRs against A375 cells; (**d**) uptake of the GNRs measured for A375 cells. Error bars represent the standard deviation of three experiments.

It is known from References [29,30,31] that plasmon resonance coupling between gold nanorods can generate large amounts of heat. In order to apply the plasmon resonance characteristics of gold nanorods to cell photothermal therapy, the optical properties and cytotoxicity of gold nanorods were studied. The modified seedless method described in Reference [21] was used to synthesize the GNRs employed in this paper. The short diameter of the GNRs was 7 ± 1.3 nm and the long diameter was 27 ± 6.1 nm. The aspect ratio (ratio of the long diameter to the short diameter) was 3.75 ± 0.73. Figure 1a shows the transmission electron microscopy (TEM) images of the GNRs. It can be seen from Figure 1a that the GNRs are regular in shape and uniform in size. Figure S1 show the GNRs average aspect ratio is 3.75, the standard deviation is 0.73. Figure 1b depicts the absorption spectra of the GNRs. The absorption peaks show that the longitudinal surface plasmon resonance (LSPR) of GNRs was located at 800 nm. Based on the plasmon resonance peak with GNRs at 800 nm, an 800-nm laser was used as excitation light in the photothermal experiment below. The cytotoxicity of GNRs to A375 cells was examined by MTT assay. In this case, the A375 cells were incubated with the GNRs of different concentrations ranging from 26 to 130 pM. The highest cell viability was 98%, corresponding to the concentration of GNRs at 26 pM; the lowest cell viability was 81%, corresponding to the concentration of GNRs at 130 pM. From Figure 1c, it can be seen that the GNRs can be considered to be nontoxic to A375 cells, as the viability of the A375 cells still exceeds 80% at the highest concentration of the GNRs after being incubated with the GNRs for 24 h. The uptake of the GNRs for A375 cells was also studied in order to realize efficient photothermal therapy. As shown in Figure 1d, it was found that the uptake of the GNRs and the concentration of the GNRs do not exhibit a linear relationship. When the culture concentration of GNRs was 78 pM, the cellular uptake of GNRs was 1360, which was the highest among the numerous culture concentrations. In order to achieve effective photothermal therapy, a culture medium with a concentration of 76 pM GNRs was used in the following experiments, because of the high viability and high uptake at this concentration.

### 3.2. Interaction between the GNRs and A375 Cells

**Figure 2:**
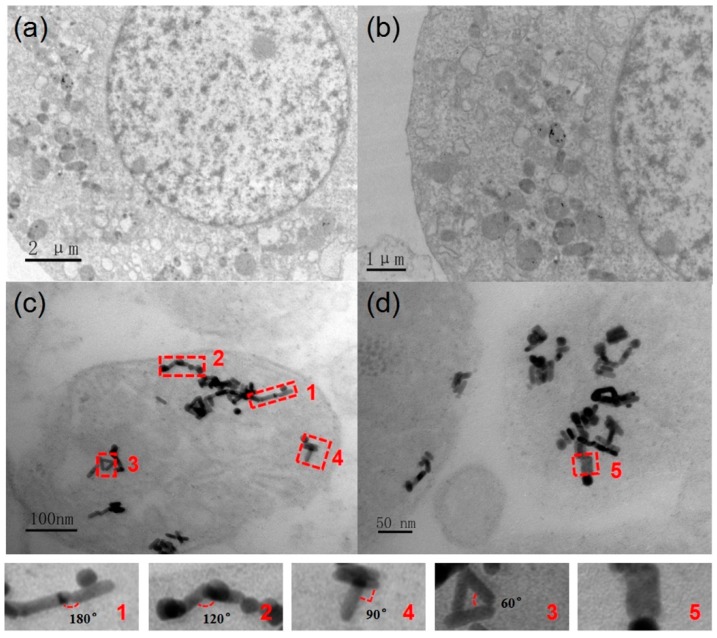
(**a**) and (**b**) are TEM images of an A375 cell that has been cultured with GNRs for 24 h (**c**) and (**d**) are TEM images of GNRs cluster naturally created in the lysosome of the A375 cell.

In order to observe the cellular uptake and localization of GNRs in A375 cells, the TEM measurements of cells were preformed after incubating the cells with GNRs for 24 h. As shown in Figure 2a, some nanoparticles were found in intracellular vesicular organelles such as lysosomes. Figure 2b–d show the TEM images of the cells incubated with GNRs for 24 h at different resolutions. The energy dispersive X-ray spectroscopy (EDX) results revealed that these nanoparticles in the cells were GNRs (see Figure S4). The other element peaks observed in the EDX spectrum come from the Ni mesh with a carbon support film and from the dyes used in the cell. Combined with information from TEM images and EDX results, it was confirmed that after 24 h of co-culture with A375 cells, GNRs were taken up by cells and accumulated in vesicular organelles after entering into cells. The GNR clusters formed naturally in lysosomes. From the TEM image of the cells, it can be seen that the gold nanorods in the vesicular organelles are at various angles with each other. Some dimers of GNRs are labeled in Figure 2c,d, and the enlarged view of these dimers of GNRs show that the angles between the gold nanorods vary from 0° to 180°.

### 3.3. The Field Enhancement of GNRs

**Figure 3:**
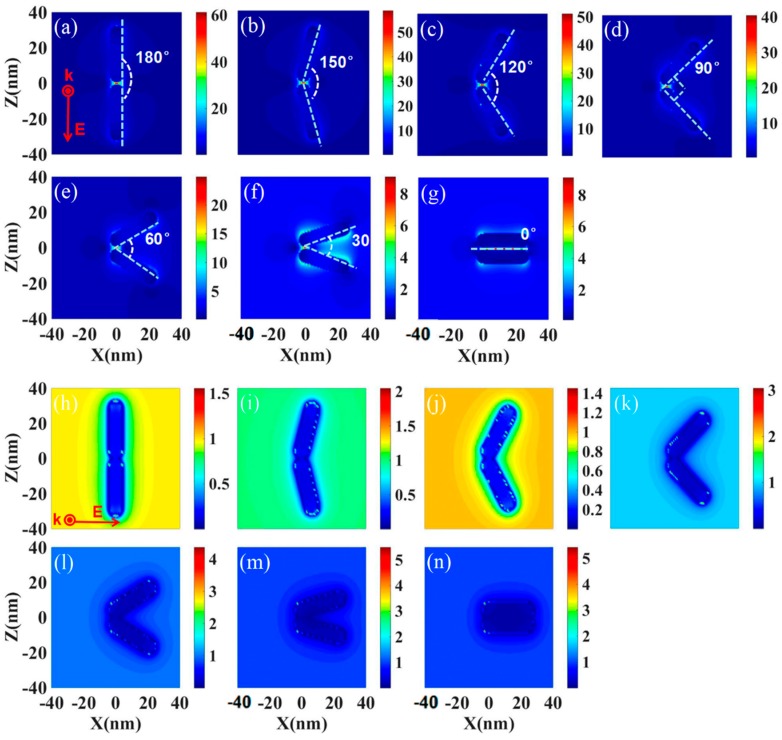
The near-field intensity map of two gold nanorods systems (LSPR 800 nm) at 800 nm laser excitation; the interparticle separation is 1 nm and the angle between the two nanorods varies from 0 degrees to 180 degrees. Both z and x polarization are studied. Images (**a**–**g**) show the z polarization; images (**h**–**n**) show the x polarization.

If the GNRs are excited by an NIR laser, upon surface plasmon formation, nonradiative relaxation occurs through electron–phonon and phonon–phonon coupling, efficiently generating localized heat that can be transferred to the surrounding environment. The photothermal conversion heat in the experiment may correspond to the field enhancement around the particles. Based on the spacing and angles of the gold nanorods in the organelles labeled in Figure 2, we performed a numerical simulation to clarify the electric field enhancement around the GNRs. Figure 3 shows the near-field intensity map of two gold nanorod systems (LSPR 800 nm) at 800 nm laser excitation. An interparticle separation of 1 nm was considered for the calculation, which is in agreement with the length of the TEM image. The field enhancement (|E|/|E_0_|) surrounding the GNRs for both z and x polarization was studied. In the case where the incident light is polarized in the z-direction, when the two gold rods are at a 180-degree angle and the pitch is 1 nm, the field-strength between the gold nanorods is as high as 60 times. This means that the laser intensity |E|^2^ at this position will increase by 3600 times. If the angle between the two GNRs changes, the field enhancement value will vary from 50 to 58. For the case where the incident light is polarized in the x-direction, when the two gold nanorods are at 0 degrees, the field enhancement will reach a maximum of 5 times, and at 120 degrees, it will be a minimum of about 1.4 times. The minimum field enhancement of 1.4 times corresponds to a laser intensity enhancement of 1.96 times. Thus, no matter which polarization direction of incident light, after interacting with the gold nanorods, it will cause field enhancement due to the plasmon resonance coupling, which will generate heat to increase the temperature of the cells and cause necrosis. It can be seen that if the laser can be aimed at the GNR clusters naturally formed in the organelles, the generated heat can achieve single-cell ultra-low energy photothermal therapy.

### 3.4. The Photothermal Therapy of GNRs

After analyzing the numerical simulation results, we conducted a series of experiments to study the interaction between the laser and the cells incubated with the GNRs. After incubating A375 cells with the GNRs for 24 h, the GNRs entered into cells and accumulated in the organelles (such as lysosomes) of the cells and formed GNR clusters. The position of the gold nanorod cluster in the cell could be obtained by scanning the cell with an 800-nm laser and observing the two-photon-absorption induced luminescence (TPL) emission image of the cell. The power of the laser used to scan the cells was below the cell’s damage threshold. After determining the location of the GNR cluster, a high-power femtosecond laser could be used to simply excite the GNR clusters. In this way, an accurate and rapid injury to a single A375 cell can be easily achieved.

**Figure 4:**
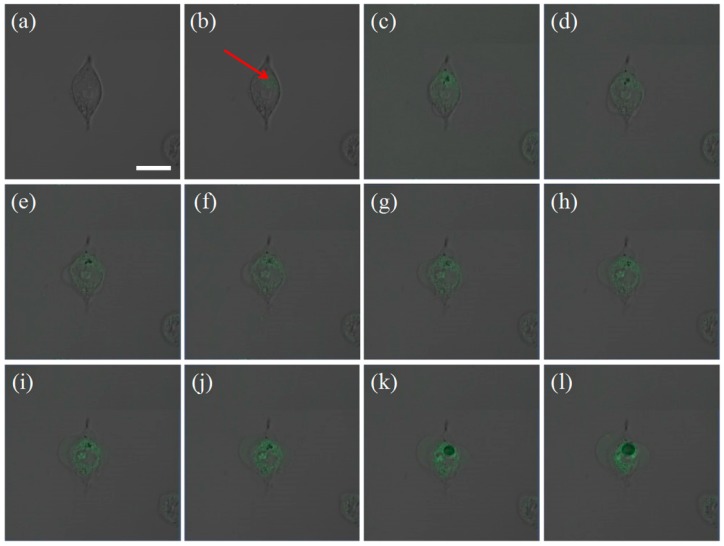
The image of an A375 cell incubated with GNRs for 24 h. (**a**) The bright field image before the laser treatment; (**b**) the combination of the bright field image and two-photon-absorption induced luminescence (TPL) emission image of the cell before laser treatment; (**c**–**l**) evolution of the cell morphology and TPL image of the excited GNR cluster when the cell was exposed to the fs laser light for different time periods of 0.1, 0.2, 0.3, 0.4, 0.5, 0.6, 0.7, 0.8, 0.9, and 1.0 s, respectively. It can be seen that afer laser treatment bubbles appears near the point of action ,with the accumulation of action time the bubbles in the cells became larger. The length of the scale bar is 20 μm.

**Figure 5:**
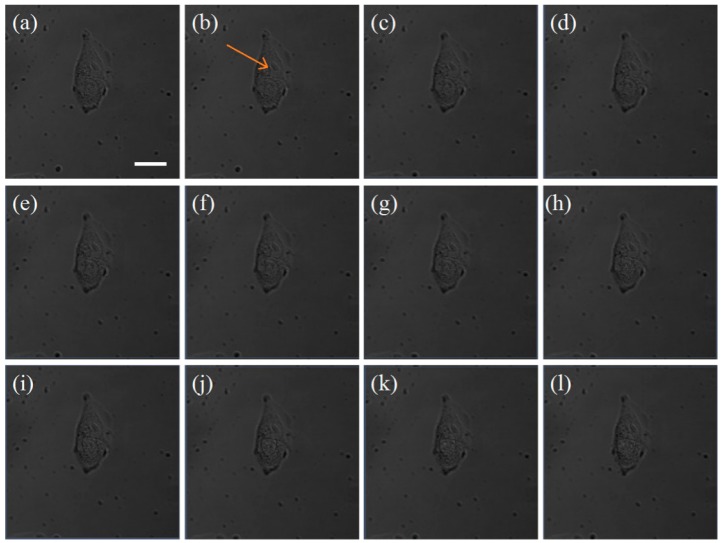
The image of an A375 cell not incubated with GNRs for 24 h. (**a**) The bright field image before the laser treatment; (**b**) the combination of the bright field image and TPL emission image of the cell before laser treatment; (**c**–**l**) evolution of the cell morphology and TPL image of the excited GNR cluster when the cell was exposed to the fs laser light for different time periods of 0.1, 0.2, 0.3, 0.4, 0.5, 0.6, 0.7, 0.8, 0.9, and 1.0 s, respectively. The length of the scale bar is 20 μm.

Figure 4 shows the process of laser treatment to a single A375 cell. Figure 4a is the bright field image of an A375 cell incubated with GNRs for 24 h. This cell was scanned with a laser with a power of 4 mW, and a two-photon emission image of the cell was obtained. Figure 4b shows the combination of the image of the bright field and the TPL emission image. The location of GNR clusters can be found by confirming the TPL emission area (indicated by the arrow in Figure 4b). Then, a high-power laser (30 mW) was use to illuminate the point indicated by the arrow in Figure 5b. The laser does not scan when it hits this point. After the laser lasts for 0.1 s, a 4-mW laser was used to rapidly scan the cell to obtain its morphology and two-photon fluorescence images. This makes it easier to study the series of changes that occur after the laser is applied to the gold nanorod clusters. From Figure 4c–l, the morphological changes of cells under different laser irradiation times are shown. From Figure 4, it can be seen that the laser light is applied to the cells for the first treatment period (0.1 s), and relatively small bubbles appear near the point of action. With the accumulation of action time, up until the ninth treatment period (0.9 s), the bubbles in the cells became larger. According to the theoretical calculation, the plasma-resonant coupling of the gold nanorods at different angles to each other can enhance the photoelectric field by 1.5–60 times. GNR ensembles act as nano-lenses that are able to confine light at subwavelength dimensions, giving rise to electromagnetic field enhancements that are dozens of times of magnitude larger than those of the incident field, known as hot spots. The temperature near a hot spot rapidly rises. The organelles in the cells are expanded by the inflation gas generated by the increasing temperature to form bubbles. The intensity and distribution of the TPL changes during the laser irradiation treatment of cells, which will be discussed in detail the following sections. If the laser light is blocked from reaching the cells at this time, the bubbles will gradually disappear, but this process of generating bubbles can damage the organelles and cause the cells to become apoptotic within a certain period of time.

For comparison, a two-photon fluorescence scan at low power on cells that had not been incubated with GNRs was performed (Figure 5), and no two-photon fluorescence was found. Then, a randomly selected position in the cell was irradiated with a high-power (30 mW) laser for 1 s (Figure 5c–l). There was no change in cell morphology, indicating that the optical power acting on the cells was not sufficient to cause damage to the cells. Comparing Figure 4 with Figure 5, we found that the hot spots generated by the coupling of intracellular gold nanorod clusters are the key factors for the photothermal treatment of a single cell within a short period of time.

**Figure 6:**
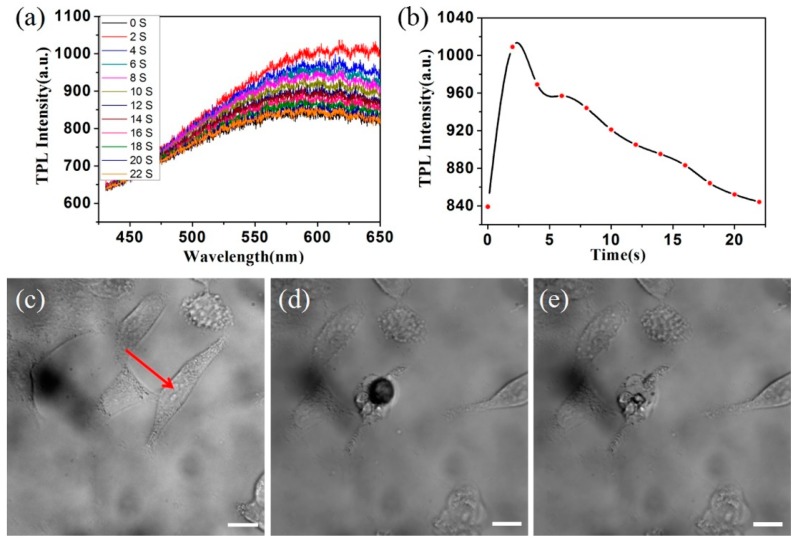
(**a**) Evolution of the TPL spectrum of the excited GNR cluster during laser treatment; (**b**) spectral integral intensity evolution of the TPL spectrum of the excited GNR cluster during laser treatment; A75 cells morphology before (**c**), during (**d**), and after (**e**) laser treatment. The length of the scale bar is 20 μm.

In order to further study the interaction between the laser and gold rods in cell culture, the cell luminescence spectra of the entire process of intracellular photothermal therapy were recorded. Figure 6c shows a single cell before laser irradiation. Figure 6d is a photograph of a cell with a laser focused onto the gold nanorod clusters. Figure 6e is an image of the cells after laser irradiation and it can be seen that the cell morphology has changed. In this process, the laser focused on the GNR clusters in the cell for 22 s, and the spectral changes in this process were recorded. Figure 6a is the two-photon fluorescence emission spectra of the cells irradiated by an 800-nm laser for 22 s. The spectral shape was similar to that of the GNRs. Figure 6b is the integration result of the spectra of Figure 6a, showing a graph of the intensity of the spectrum of a cell as a function of time during the bubble-forming process of a cell by laser excitation. It can be seen from Figure 6b that, when the laser just started irradiating the organelles containing GNR clusters in the cells, the bubble appeared. This is due to the increasing temperature inside the organelles. The field enhancement caused by the coupling of plasmon resonances between the GNRs led to this temperature increase. With the generation of the bubble, the intensity of the spectrum was greatly enhanced. As the bubble volume increased first and then decreased, the spectral intensity also decreased from strong to weak. This is a complex biophysical process. There are some possible reasons for this increase in TPL strength: first, the plasma coupling effect between the gold nanorods makes the fluorescence intensity increase; second, the organelle is thermally expanded to make the cell scattering cross-section larger; third, during the laser action process, some of the organelles change due to the heat.

**Figure 7:**
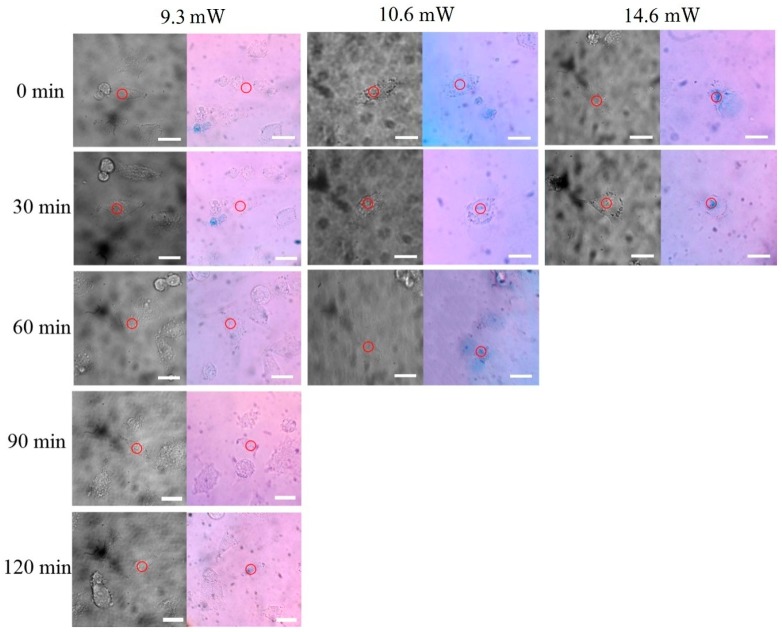
Bright field images of the cells on which the laser treatment was carried out. The color images show the cells dyed with Trypan Blue after the laser treatment. Laser light with different laser powers was employed in the laser treatment experiments. The cells after the irradiation of the laser light were dyed with Trypan Blue after different intervals of time of 0, 30, 60, 90, and 120 min. The blue color appearing in some areas without dead cells is caused by Trypan Blue, which did not diffuse uniformly in the experiment. The length of the scale bar is 20 μm.

The above experimental results demonstrate that the photothermal treatment of cells can be achieved with low power using the plasma resonance coupling effect of GNR clusters. In order to further study the photothermal effect of GNRs in cells, different laser intensities were used to excite the GNRs in cells to achieve the purpose of regulating the death time of cells. Figure 7 shows the excitation of gold nanorods with different power lasers. As can be seen from Figure 7, with the 14.6-mW, laser-focused irradiation of organelles containing GNR clusters for 20 s, cells can immediately undergo necrosis. If the cells are irradiated by a 10.6-mW laser for 20 s, they do not undergo necrosis immediately, but will die after 60 min. If the cells are irradiated by a power of 9.3 mW laser for 20 s, they will die after 120 min. The results of Figure 7 are summarized in Table 1.

## 4. Conclusions

In conclusion, a fast photothermal therapy of single A375 cells was investigated in this paper. After incubation with GNRs for 24 h, the cell viability of A375 cells exceeded 80%. The cellular uptake of gold nanorods reached as high as 1360 GNRs per cell. These two conditions make gold nanorod photothermal therapy easier to develop for A375 cells. From the theoretical calculation results, it was revealed that the plasmon resonance coupling between the gold nanorods can lead to a field enhancement up to 60 times. This field enhancement leads to a larger temperature rise, which can be used to induce the death of single cells in a short time and under low laser power excitation. The position of the GNR clusters can be identified by observing the TPL images of A375 cells. Laser excitation of GNR clusters leads to changes in cell morphology and bubble generation, leading to necrosis in irradiated cells. The deaths time can be controlled by changing the input laser power. Our results help to achieve a small range of low-energy photothermal therapy, which will open up a new path for the application of nanoparticles in biomedicine.

## Figures and Tables

**Table 1:** Summary of the information about the laser power and cell death time.

**Power**	14.6 mW	10.6 mW	9.3 mW
**Cell Death Time**	after 0 min	after 60 min	after 120 min

## Supplementary Materials

The following are available online at http://www.mdpi.com/2079-4991/8/11/880/s1. Figure S1: (a) The TEM image of the GNRs; (b) the statistical analysis of the GNRs, the average aspect ratio is 3.75, the standard deviation is 0.73; Figure S2: Shapes of A375 cells observed using an inverted light microscope after exposure to GNRs at a concentration of (a) 78 pM, (b) 104 pM, and (c) 0 pM (without GNRs) for 24 h; Figure S3: Experimental setup of photothermal therapy; Figure S4: The energy dispersive spectroscopy measurements of GNPs found in the TEM image of the vesicle, verifying the existence of Au element; Inset: The mass ratio of each element.
