# Supplementary material for: A Novel Fast Photothermal Therapy Using Hot Spots of Gold Nanorods for Malignant Melanoma Cells
Source: Nanomaterials (Basel). 2018 Oct 28;8(11):880. doi: 10.3390/nano8110880 (PMC6266339; doi:10.3390/nano8110880)
Supplement: Supplementary file 1 [file nanomaterials-08-00880-s001.pdf]

Supporting Information

A Novel Fast Photothermal Therapy Using Hot Spots of Gold Nanorods for Malignant Melanoma Cells

Yan-Hua Yao [1], Nan-Nan Zhang, Xiao Liu [1], Qiao-Feng Dai [1], Hai-Ying Liu [1], Zhong-Chao Wei [1], Shao- Long Tie [2],Yin-Yin Li [3], Hai-Hua Fan [1,*], Sheng Lan [1,*]

1 Guangdong Provincial Key Laboratory of Nanophotonic Functional Materials and Devices, School of Information and Optoelectronic Science and Engineering, South China Normal University, Guangzhou 510006,China

2 School of Chemistry and Environment, South China Normal University, Guangzhou 510006, China

3 School of Life Sciences, Sun Yat-Sen University, State Key lab for biocontrol，Guangzhou Guangdong 510275,China。

* Correspondence: fhh12345@163.com (H.F.); slan@scnu.edu.cn (S.L.); Tel.: +86-20-3931-0309

1. Synthesis of Polyethylene Glycol (PEG)-Coated Gold Nanorods (GNRs)

At the temperature of 28−30℃, 5 ml of 0.2 mM cetyltrimethylammonium bromide (CTAB) was added to 5 ml of 1 mM $HAuCl_4$ H_2O solution, and 300 μL of 4 mM $AgNO_3$ was added gradually. The pH value of the solution was regulated to 1−1.15 by hydrochloric acid, and then 70 μL of 78.8 mM ascorbic acid was added. The solution gradually became colorless and the stirring was stopped. Immediately afterward, ice-cold NaBH4 (15 μL, 0.01 M) was injected into the unstirred growth solution and allowed to react for 6 h. The amount of AgNO3, hydrochloric acid, and $NaBH_4$ in the reaction was controlled to adjust the size of gold nanorods and the position of absorption peaks in the near-infrared range. After the reaction was completed, the free CTAB was centrifugally removed and washed three times, and then dispersed in water. The as-prepared GNRs were then stabilized by polyethylene glycol (PEG).

Figure S1 shows the TEM image of GRNs and a statistical analysis of the aspect ratio of GNRs. The average diameter of the GNRs was 7 nm and the average length was 27 nm. The average aspect ratio was 3.85 and the standard deviation was 0.73.

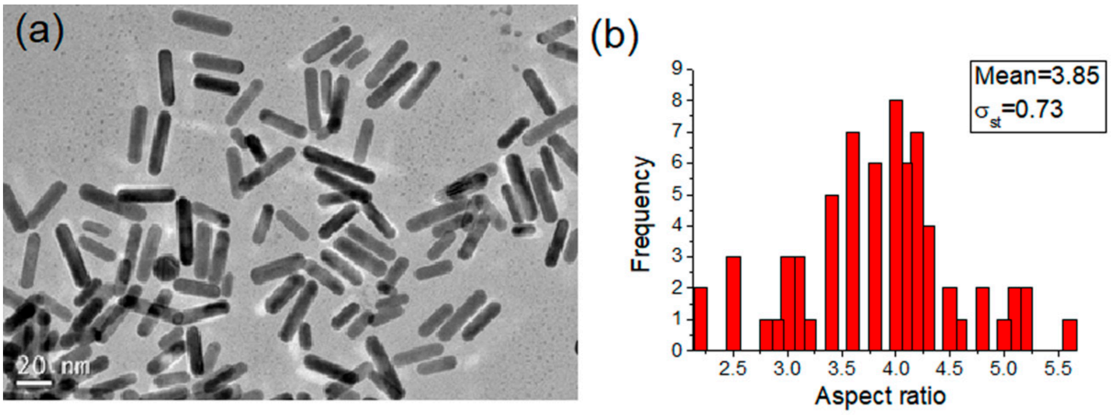

Figure S1. (**a**) The TEM image of the GNRs; (**b**) the statistical analysis of the GNRs. The

average aspect ratio is 3.75; the standard deviation is 0.73.

2. Measurement of Cell Viability

A375 cells were cultured in a humidified incubator at 37 °C with an atmosphere containing 5% CO_2. The culture medium was composed of 89% Dulbecco's Modified Eagle Medium (DMEM), 10% fetal bovine serum, and 1% penicillin/streptomycin. In all experiments concerning the viability of cells described in this work, the MTT assay was employed to determine the viability of cells. Apart from the MTT assay, we also examined the shapes of cells, from which the apoptosis of cells can be identified, by using an inverted light microscope. In Figure S1, the shapes of A375 cells after incubation without GNRs and after incubation with GNRs with different concentrations for 24 hours are shown.

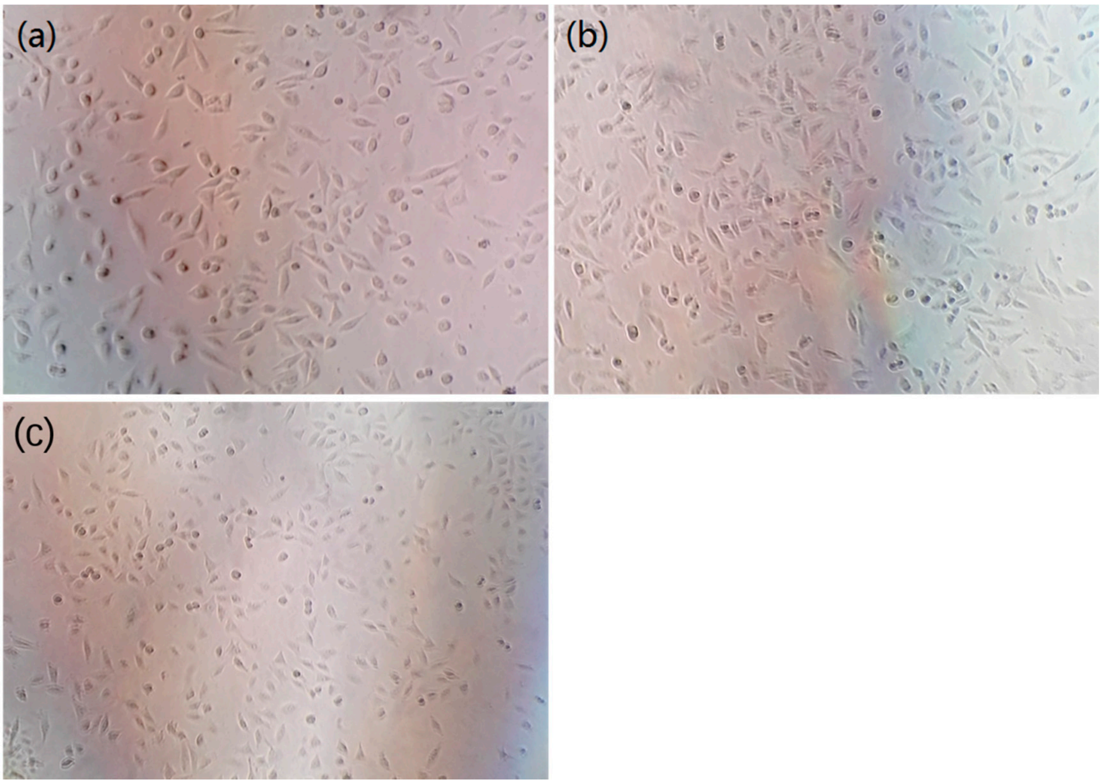

Figure S2. Shapes of A375 cells observed using an inverted light microscope after exposure to GNRs at a concentration of (**a**) 78 pM, (**b**) 104 pM, and (**c**) 0 pM (without GNRs) for 24 h.

3. Experimental Setup for the Laser Treatment of Cells

The experimental setup used for the laser treatment of cells is schematically shown in Figure S2.

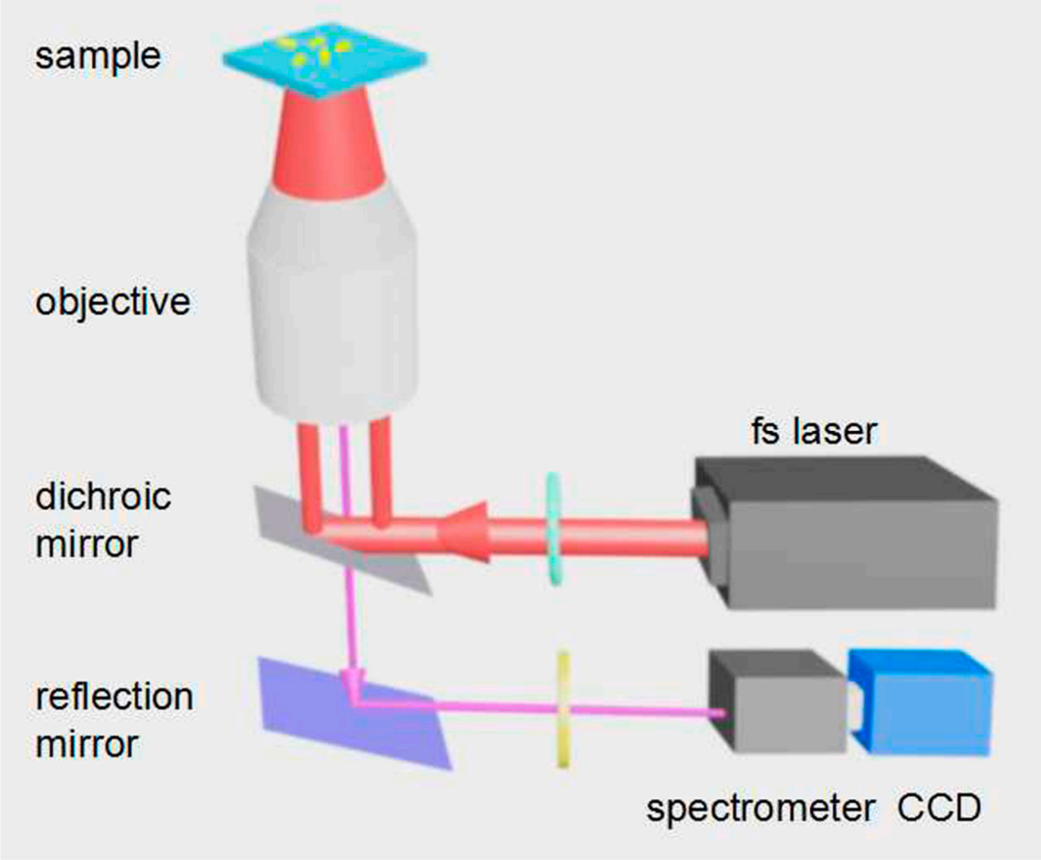

Figure S3. Experimental setup of photothermal therapy.

3. Interaction between the GNRs and A375 Cells

After being incubated with GNRs for 24 hours, we examined the cells by TEM measurements and found some nanoparticles located in intracellular vesicular organelles such as lysosomes. The energy dispersive spectroscopy is shown in Figure S3.

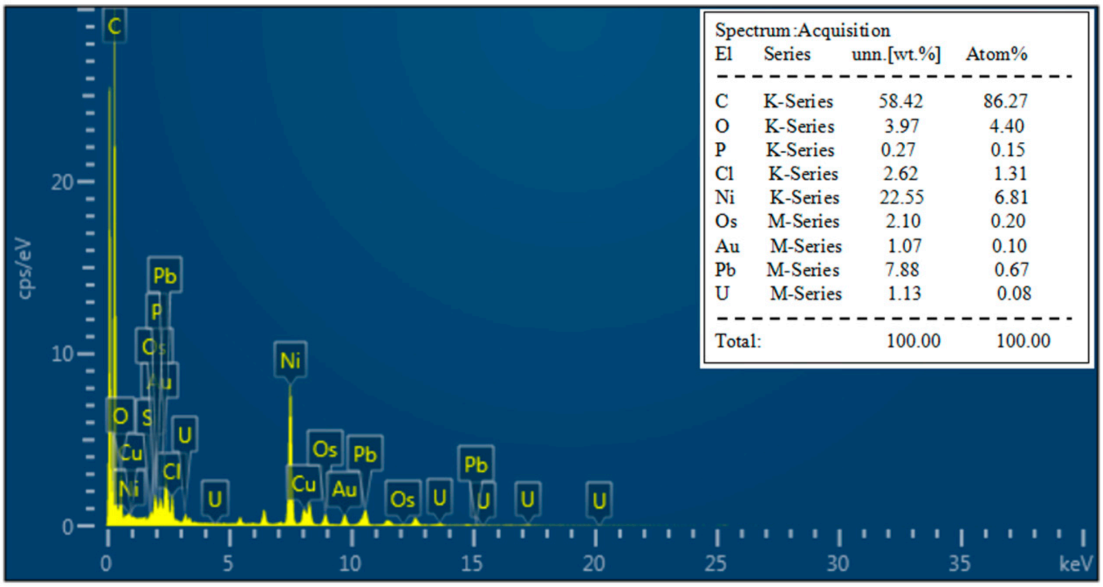

Figure S4. Energy dispersive spectroscopy measurements of GNPs found in the TEM image of the vesicles, verifying the existence of Au element. Inset: The mass ratio of each element.
